# The role of dupilumab in skin microbiome shifts in the Netherton genodermatosis: a case report and review of literature

**DOI:** 10.3389/fmed.2026.1854027

**Published:** 2026-06-19

**Authors:** Elena Campione, Anna Simonelli, Enrico Salvatore Pistoia, Terenzio Cosio, Fabio Artosi, Laura Diluvio, Luca Bianchi, Roberta Gaziano

**Affiliations:** 1 Dermatology Unit, Department of Systems Medicine, Tor Vergata University Hospital, Rome, Italy; 2 Otorinolaryngology Unit, Department of Sense Organs, Policlinico Umberto I University Hospital, Rome, Italy; 3 Department of Experimental Medicine, University of Rome Tor Vergata, Rome, Italy; 4 Department of Basic Biotechnological Sciences, Intensive and Perioperative Clinics, Università Cattolica del Sacro Cuore, Rome, Italy; 5 Department of Biochemical Sciences, Catholic University "Our Lady of Good Counsel", Tirana, Albania

**Keywords:** case report, dupilumab, genodermatosis, Netherton syndrome, NGS, skin microbiome

## Abstract

Skin dysbiosis plays a crucial role in inflammatory skin diseases, particularly in genodermatoses such as Netherton syndrome (NS). This case report aimed to investigate changes in the skin microbiome of a patient with Netherton syndrome before and during dupilumab therapy, with the goal of expanding the limited evidence currently available on this topic. We report the case of a 35-year-old woman diagnosed with NS at birth, who, prior to dupilumab therapy, presented with atopic dermatitis (AD), ichthyosis linearis circumflexa, and severe pruritus. Dupilumab therapy was initiated, and skin swabs were collected from lesional sites at three different time points: at baseline, after 1 month, and after 1 year of continuous dupilumab therapy. At baseline, a microbiome analysis revealed low microbial diversity with a predominance of *Pantoea* and *Pseudomonas* species. After 1 year, a significant increase in microbial diversity, with a predominance of *Staphylococcus* species and an increase in *Malassezia* species, was observed. Clinically, the patient experienced remission in parallel with these microbiome shifts. Post-treatment, the skin microbiome showed increased microbial diversity and re-establishment of beneficial commensals, more closely resembling healthy skin. The findings of this case report underscore the role of dupilumab in restoring a healthy skin microbiome along with symptomatological and clinical improvement in the genodermatosis Netherton syndrome.

## Introduction

Netherton syndrome is a rare autosomal recessive genodermatosis characterized by congenital ichthyosis, hair shaft abnormalities, and atopy (1, 2). It is caused by mutations in the *SPINK5* gene, which encodes the serine protease inhibitor lympho-epithelial Kazal-type-related inhibitor (LEKTI). The loss of LEKTI function leads to the unopposed activity of kallikreins (KLKs) 5, 6, 7, 13, and 14, which disrupts the skin barrier integrity, causing premature detachment of the stratum corneum and driving Th2-mediated inflammation. This inflammation is characterized by increased interleukin (IL)-4, IL-5, and IL-13 release; high serum immunoglobulin E (IgE) levels; and hypereosinophilia (3).

Ichthyosiform erythroderma, a hallmark of NS, can evolve into ichthyosis linearis circumflexa, a highly specific, although not consistently present, manifestation of the condition (3). Trichorrhexis invaginata, also known as “bamboo hair,” is another distinctive clinical sign (4). A constant feature of NS is the presence of multisystem atopic manifestations, including eczematous-like skin lesions, allergic rhinitis, conjunctivitis, asthma, and angioedema (5).

Recent studies have highlighted an association between NS and skin microbiome dysbiosis. Patients with NS commonly show increased colonization by *Staphylococcus aureus* and *Staphylococcus epidermidis*, both of which produce proteases that exacerbate barrier dysfunction (6). Moosbrugger-Martinzet et al. reported a reduction in microbial diversity and a predominance of *Firmicutes* (particularly *Staphylococcus*) and *Actinobacteria* (mainly *Corynebacterium*) on NS skin. Additionally, they observed alterations in fungal communities, most notably an overrepresentation of *Cladosporium*, which was associated with elevated serum IgE. A reduction in beneficial commensals, such as *Clostridia* and *Lactobacillus* species, was also observed (7). Further studies have confirmed similar patterns of dysbiosis, with variability in the dominant fungal species. One report identified an overabundance of *Malassezia globosa* and *Malassezia restricta*, whereas *Cladosporium* was less prominent (8). Another study by Tham et al. (9) described reductions in key commensals such as *Cutibacterium* and *Staphylococcus hominis*, along with increases in *Staphylococcus* and *Corynebacterium* genera.

Despite the severity of NS, no standardized management guidelines currently exist (4). Topical corticosteroids are commonly used, and topical calcineurin inhibitors have also demonstrated efficacy in reducing the clinical severity of NS (3, 10, 11). In addition, a case report has documented successful management using narrowband ultraviolet B (NB-UVB) phototherapy (12). However, topical therapies are often insufficient, and systemic agents are frequently required for adequate disease control (4). Among systemic treatments, retinoids have been widely reported in the management of NS with variable efficacy (4, 13). More recently, immunoglobulin therapy and biologic agents targeting specific inflammatory pathways have emerged as promising treatment strategies (4, 14). In particular, several studies have highlighted the use of biological molecules targeting IL-17, IL-23, IL-4 receptor (IL-4R), and IL-13R, as well as small molecules such as Janus kinase (JAK) inhibitors (14, 15). Notably, Sun *et al.* reported successful treatment with upadacitinib, a JAK-1 inhibitor, in a 14-year-old boy with NS (16). Case reports have also demonstrated effective management of the atopic manifestations of NS using biologics that inhibit IL-4R and IL-13R (17–19).

Dupilumab, a monoclonal antibody targeting the IL-4 receptor *α* subunit, has been shown to be effective in managing the cutaneous manifestations of NS (20). Several case reports have documented clinical improvement following dupilumab therapy, including reductions in pruritus and scaling, although variable responses have been noted (17, 21, 22). Additionally, dupilumab has been associated with increased microbial diversity and reduced *Staphylococcus aureus* colonization in other inflammatory skin conditions (23).

To date, however, only one published study has explored the effect of dupilumab on the skin microbiota of a pediatric patient with NS (24).

Given the limited data on the impact of dupilumab on the skin microbiome in NS, this case report describes changes in the skin microbiome of an adult woman diagnosed with NS at baseline, 1 month after, and 1 year after dupilumab treatment. Particular attention is given to the relationship between clinical improvement and shifts in the microbial community.

## Case presentation

**Figure 1:**
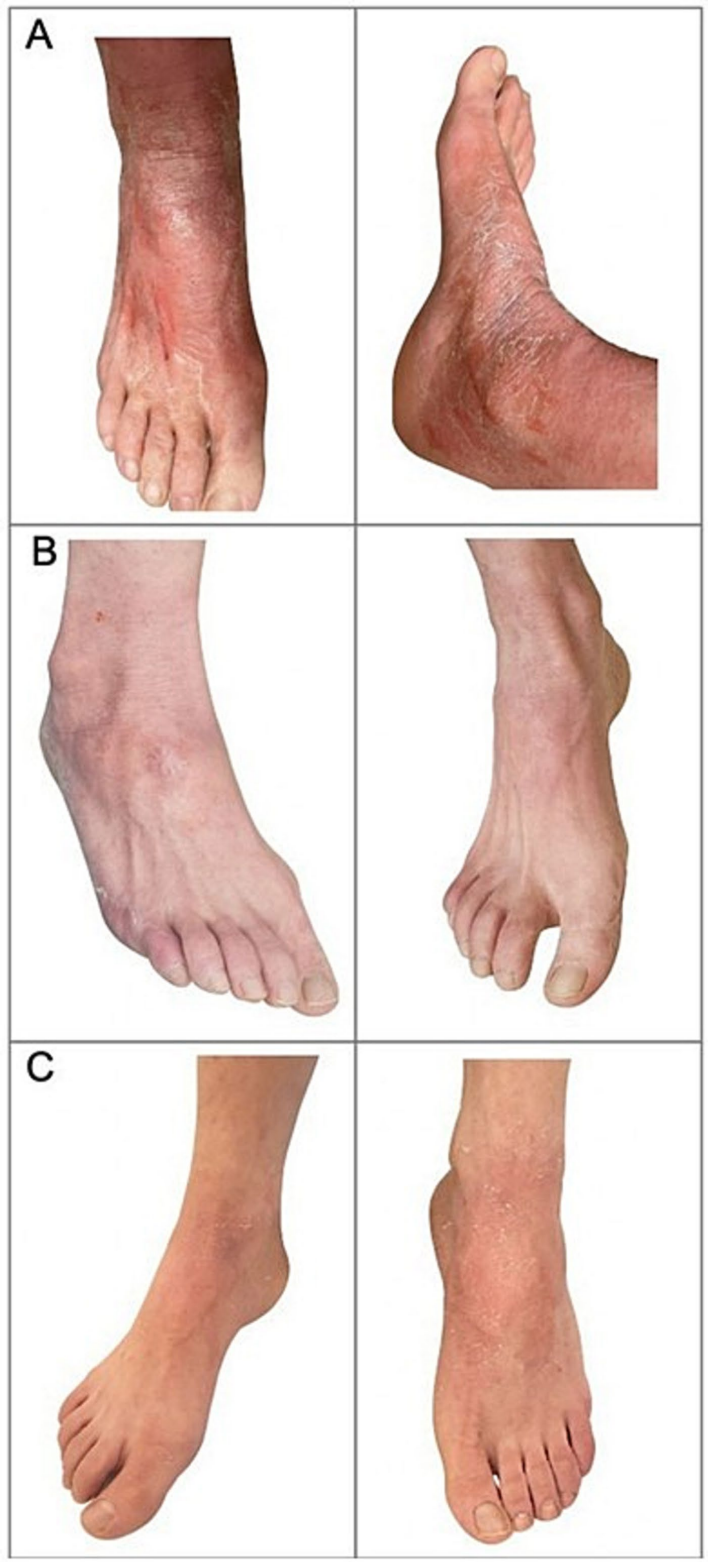
Clinical presentation of the patient’s right foot during dupilumab treatment. At baseline **(A)**, significant erythema and desquamation were observed, consistent with active skin inflammation. Following the initiation of dupilumab treatment, a progressive improvement was observed at T1 **(B)**, with a near-complete resolution of erythema and scaling at T2 **(C)**.

We report the case of a 35-year-old woman diagnosed with a SPINK5 gene mutation diagnosed at birth who presented to our department. She has had atopic dermatitis since birth. Her symptoms had previously been treated with antihistamines and topical corticosteroids, with only temporary improvement. The patient’s clinical condition worsened after her first pregnancy, presenting with ichthyosis linearis circumflexa, widespread erythroderma, and desquamation, along with intolerable pruritus (Figure 1A). The patient also expressed concern regarding her physical appearance as a consequence of the dermatological manifestations of Netherton syndrome.

Dupilumab therapy was initiated (loading dose of 600 mg, 300 mg every 2 weeks). Dupilumab was well tolerated by the patient, who did not report or present adverse or unanticipated events during the treatment. The patient maintained good adherence to the treatment. Adherence and tolerability were assessed during regular follow-up visits based on patient interviews and physical examination.

Skin swab samples were collected from the dorsum of the right foot, the skin area exhibiting the most pronounced signs of inflammation, at three time points: baseline (prior to dupilumab initiation, T0), 1 month after starting treatment (T1), and 1 year after starting treatment (T2). Sampling was performed using eSwab™ collection devices (Copan, Italy). At every follow-up, the clinical scores used were the Eczema Area and Severity Index (EASI) score, which is a validated tool used to assess the extent and severity of atopic dermatitis. Numerical Rating Scale (NRS) score for pruritus, and the NRS score for sleep disturbance. The nucleic acids from the swabs were extracted using the Molgen Universal Extraction Kit (Adaltis, S.r.l, Milan, Italy), according to the manufacturer’s instructions. Briefly, the swabs were incubated in lysis buffer to disrupt microbial cell membranes and release nucleic acids. Following chemical lysis, nucleic acids were purified using the automated/silica-based extraction workflow provided by the kit, including washing and elution steps to obtain purified DNA suitable for downstream sequencing applications. DNA concentration and purity were assessed prior to library preparation.

Skin microbiome profiling was performed using the CE-IVD-certified Microbiome Plus Panel Long Kit (4bases, Manno, Switzerland), specifically designed for long-read sequencing of bacterial and fungal targets on the Oxford Nanopore Technologies platform (25). The assay enables amplification of taxonomic marker genes, including bacterial 16S rRNA regions and fungal ITS targets, allowing simultaneous characterization of bacterial and fungal communities. Library preparation was carried out according to the manufacturer’s protocol, including target amplification, barcoding, and adapter ligation. Sequencing data were generated in QFAST format and analyzed using the One Codex platform for taxonomic classification. Microbial diversity was assessed using alpha diversity metrics, including the Simpson index, the Shannon index, and species richness.

At baseline (T0), the patient had recently completed a pregnancy and reported worsening of skin symptoms, including increased pruritus and erythema. Clinical assessment revealed the following scores: Eczema Area and Severity Index (EASI) score: 25; Numerical Rating Scale score for pruritus (NRS): 10; NRS score for sleep disturbance: 10 (Table 1). Moreover, on clinical examination, significant erythema and desquamation were observed on the patient’s right foot, consistent with active skin inflammation (Figure 1A).

**Table 1:** Changes in EASI, NRS pruritus, and NRS sleep scores during dupilumab treatment.

Clinical score	T0	T1	T2
EASI	25	15	10
NRS pruritus	10	5	2
NRS sleep	10	5	0

**Figure 2:**
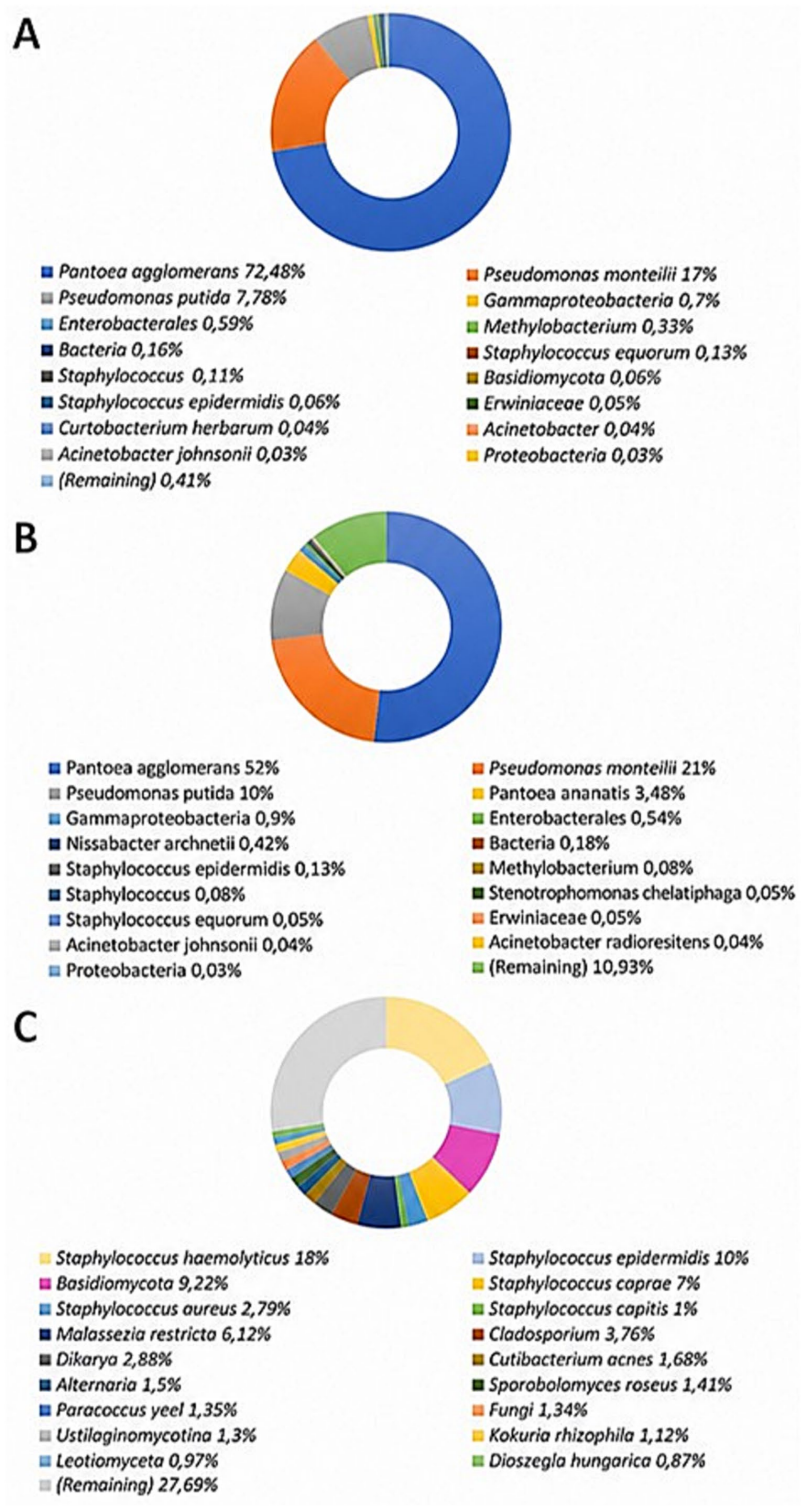
Changes in the skin microbiota composition of a patient with NS during dupilumab treatment. At T0 **(A)**, the skin microbiota was dominated by Pantoea agglomerans, with Pseudomonas spp. as the second most abundant group. At T1 **(B)**, Pantoea decreased while Pseudomonas increased, although both remained dominant. At T2 **(C)**, microbial diversity increased, with a shift toward Gram-positive bacteria dominated by Staphylococcus spp.; Cutibacterium acnes and fungal taxa including Malassezia restricta, Cladosporium, and Alternaria were also detected.

At the same time point, analysis of the patient’s cutaneous microbiome revealed a distinct microbial profile, dominated by Gram-negative bacteria. As shown in Figure 2A, the skin microbiome was heavily dominated by *Pantoea agglomerans*, which accounted for more than 70% (72.48%) of the identified bacterial species.

In addition to *Pantoea*, the remaining bacterial community consisted primarily of *Pseudomonas* spp. (24.78%), of which *Pseudomonas monteilii* and *Pseudomonas putida* accounted for approximately 17 and 7.78%, respectively.

At the 1-month follow-up (T1), the patient’s clinical parameters improved. The EASI score had decreased to 15, while the NRS score for pruritus and the NRS score for sleep were both reduced to 5 (Table 1). Figure 1B shows the clinical appearance of the patient’s right foot at T1, demonstrating a visible improvement compared with the baseline.

Interestingly, as shown in Figure 2B, microbiome analysis at T1 revealed a moderate modification in microbial composition compared to the baseline (T0). While the microbiota remained dominated by *Pantoea* and *Pseudomonas* species, their relative abundances changed over time. Specifically, *Pantoea* spp. decreased from 72.48% at T0 to 55.48% at T1, whereas *Pseudomonas* spp. increased from 24.78 to 41.47%. Within the *Pseudomonas* genus, *P. monteilii* and *P. putida* were the most represented species at this time point, comprising 21 and 10% of the total community, respectively, with *P. monteilii* maintaining its predominance within the *Pseudomonas* species.

The patient remained on continuous dupilumab therapy throughout the year. At the 1-year follow-up (T2), a sustained and significant clinical improvement was observed, with an EASI score of 10, an NRS pruritus score of 2, and complete resolution of sleep disturbance (NRS score: 0). A significant improvement in skin condition was also noted, with near-complete resolution of erythema and scaling (Figure 1C).

The skin swab at T2 revealed significant changes, with a significant increase in microbial diversity and a shift toward a composition more characteristic of healthy skin. While the microbiome at baseline and at T1 was dominated by Gram-negative bacteria (*Pantoea* and *Pseudomonas* species), the T2 profile showed a notable increase in Gram-positive commensals, particularly *Staphylococcus* spp. (38.79%), including *Staphylococcus haemolyticus* (18%), *Staphylococcus epidermidis* (10%)*, Staphylococcus caprae* (7%), *Staphylococcus aureus* (2.79%), and *Staphylococcus capitis* (1%). In addition, the relative abundance of the commensal fungal yeast *Malassezia restricta* increased to 6.12%, while the relative abundance of filamentous fungi, including *Cladosporium* and *Alternaria*, reached 3.7 and 1.5%, respectively. *Cutibacterium acnes*, a key skin-beneficial bacterium, also emerged at 1.68%. Notably, all of the abovementioned fungi, as well as *Cutibacterium acnes,* were undetectable at both T0 and T1 (Figure 2C). Overall, the T2 microbiome exhibited a more balanced and evenly distributed community of skin commensals, aligning more closely with the microbial profile typically associated with healthy skin. This restoration of microbial homeostasis was further supported by a progressive increase in all three alpha diversity metrics—Simpson index, Shannon index, and richness—from baseline (T0) to 1 month (T1) and further at 1 year (T2) (Table 2). A descriptive summary of the dominant lesional skin microbiome findings across the three sampling time points is provided in Table 3.

**Table 2:** Alpha diversity parameters at the species and genus levels at baseline (T0), after 1 month (T1), and after 1 year of dupilumab therapy (T2).

Diversity parameter	T0		T1		T2	
	Species	Genus	Species	Genus	Species	Genus
Simpson index	0.41	0.39	0.65	0.50	0.91	0.71
Shannon index	1.23	0.92	2.01	1.10	4.37	3.11
Richness	17.00	15.00	21.00	18.00	134.00	126.00

**Table 3:** Longitudinal changes in dominant lesional skin microbiome composition.

Time point	Clinical status	Dominant skin microbiome composition
T0, baseline	Active disease, with marked erythema, desquamation, and severe pruritus.	Low-diversity profile dominated by *Pantoea agglomerans* (72.48%), followed by *Pseudomonas* spp. (24.78%)
T1, 1 month	Early clinical improvement, with reductions in EASI, pruritus, and sleep-disturbance scores.	Persistent predominance of Gram-negative taxa. *Pantoea* spp. decreased to 55.48%, whereas *Pseudomonas* spp. increased to 41.47%
T2, 1 year	Sustained clinical improvement, with near-complete resolution of signs and symptoms.	Increased taxonomic diversity with reduced Gram-negative dominance and the emergence of skin-associated taxa, including *Staphylococcus* spp. (38.79%), *Cutibacterium acnes* (1.68%), *Malassezia restricta* (6.12%), *Cladosporium* sp. (3.76%), and *Alternaria* sp. (1.5%)

Altogether, these findings suggest that long-term dupilumab treatment not only improves clinical symptoms but also restores microbial diversity on the skin, which may contribute to the overall therapeutic effect.

## Discussion

Our results revealed that, at baseline (T0), the patient’s skin exhibited low microbial diversity with a predominance of Gram-negative bacteria, particularly *Pantoea agglomerans* (72.48%) and *Pseudomonas* species (24.78%). The predominance of *Pantoea*, a genus typically associated with soil and plant environments, is unusual for human skin and may reflect an increased susceptibility of the compromised skin barrier to colonization by environmental bacteria. In the context of active inflammation and structural barrier defects characteristic of NS, such organisms may persist and expand, contributing to or perpetuating cutaneous dysbiosis. Although *Pantoea* species have been isolated from atopic dermatitis (AD) lesions in clinical culture-based studies, their role in skin inflammation remains poorly understood (26). Increasing evidence suggests that a healthy skin microbiome is predominantly composed of Gram-positive bacteria, with Gram-negative species typically present in much lower abundance (26, 27). The elevated abundance of *Pantoea* in this patient, together with clinical signs of active disease, suggests that *Pantoea* may either thrive in or potentially contribute to the inflamed skin environment; however, further studies are required to clarify its role. Similarly, the concurrent overrepresentation of *Pseudomonas* spp., particularly *P. monteilii* and *P. putida*, further supports the presence of an altered microbial ecosystem in which environmental organisms may persist and potentially displace resident commensals. Interestingly, *Pseudomonas* species have been reported to be more prevalent in pregnant women due to hormonal and immunologic changes during pregnancy (28). Considering that our patient had recently experienced pregnancy, this physiological state may have contributed to the observed expansion of *Pseudomonas* in her skin microbiota, potentially acting in concert with disease-related barrier dysfunction. After 1 month (T1) of dupilumab treatment, only minimal changes in the skin microbiome composition were observed, characterized by a modest reduction in *Pantoea* and an increased relative abundance of *Pseudomonas* species. Nevertheless, the patient showed a significant clinical improvement, with significant reductions in EASI and itch scores. These findings indicate that, while clinical symptoms improved, the skin microbiota did not exhibit significant compositional changes at the same rate, suggesting that dupilumab’s anti-inflammatory effects may occur independently of early microbiome shifts.

In contrast to the limited microbial changes observed at T1, 1 year after initiating dupilumab therapy (T2), the patient showed further clinical improvement accompanied by substantial restructuring of the skin microbiome. Specifically, the microbial community resembled that of healthy skin, with Gram-positive commensals, particularly *Staphylococcus* spp. (38.79%) and *Cutibacterium acnes* (1.68%), emerging alongside the fungal commensal *Malassezia re*stricta (6.12%), all of which were absent at earlier timepoints (29). These findings contrast with those of Moosbrugger-Martinez et al., who reported increased relative abundances of *Staphylococcus* in patients with NS (5, 7).

A key strength of this case report is the observation that the progressive increase in *Staphylococcus epidermidis* during dupilumab treatment from 0.06% at T0 to 0.13% at T1 and 10% at T2, as well as the emergence of *Cutibacterium acnes*, both known for their immunomodulatory and protective roles, suggests a restoration of microbial balance in the patient (30).

This observation was further supported by the increase in alpha diversity (Simpson, Shannon, and richness indices), a known hallmark of skin health. Consistent with our findings, previous studies have shown that long-term dupilumab therapy was associated with the restoration of microbial diversity and the suppression of pathogenic species such as *Staphylococcus aureus* (27). *Staphylococcus aureus*, often associated with AD exacerbations, was present at low abundance (2.79%) in the patient’s skin after 12 months of dupilumab therapy (T2), suggesting that dupilumab may selectively reduce its overgrowth while allowing re-colonization by commensal *Staphylococcus* species, such as *S. haemolyticus* (18%) and *S. epidermidis* (10%) (31). In addition to these bacterial shifts, the emergence of yeast fungi from the *Malassezia* genus, as well as filamentous fungi such as *Cladosporium* and *Alternaria*, also suggests a shift toward a more diverse skin-associated microbial community (32–34).

Overall, these findings highlight a delayed but progressive shift toward a more diverse skin-associated microbial community in response to dupilumab treatment, paralleling the patient’s clinical improvement. While inflammation control appears to precede full microbial restoration, the subsequent re-establishment of a diverse, commensal-rich microbiota may play a key role in maintaining long-term remission.

A key strength of this case report is the longitudinal analysis of the microbiome during treatment, enabling the evaluation of short-term and long-term microbiome changes. Moreover, the combined assessment of bacterial and fungal DNA provides a broader characterization of the skin microbiome. The findings of this case report are limited in their interpretation due to single-patient analysis, the absence of control samples, and the inherent limitations of DNA-based microbiome analysis. In light of our findings, further investigation into microbiome alterations in pregnant women with NS is warranted, particularly to elucidate how pregnancy-related immunological and hormonal changes may influence the composition and dynamics of skin microbial communities. Exploring the long-term stability of the skin microbiome under continued dupilumab therapy could provide valuable insights into sustained therapeutic effects, potential relapse prevention, and the role of a shift toward a more diverse skin-associated microbial community in maintaining clinical remission. Such studies could help refine therapeutic strategies and improve outcomes in this rare and complex condition.

## Conclusion

This case report provides novel insights into the interplay between skin inflammation, microbial dysbiosis, and immune modulation in a patient with NS post-pregnancy. The unusual dominance of *Pantoea* raises important questions about environmental microbial invasion during periods of immune vulnerability, such as pregnancy and the postpartum period. Future studies should explore the role of rare environmental microbes in NS and other barrier-defective skin disorders. Moreover, the observed clinical improvement preceding a shift toward a more diverse skin-associated microbial community suggests that anti-inflammatory treatment may create a permissive environment for the re-establishment of commensal microbes. Importantly, in our patient, microbiome changes paralleled a significant improvement in clinical symptoms, underscoring the dual action of dupilumab in both immunomodulation and indirect microbiome restoration. These findings highlight the potential of dupilumab not only as an effective treatment option for managing the clinical manifestations of NS but also as a therapeutic agent capable of promoting microbial rebalancing in the skin. The complex interplay between host immunity and the skin microbiome suggests that targeting inflammatory pathways may provide broader benefits beyond symptom control. Future studies, involving larger cohorts, are needed to confirm these findings and explore the mechanistic associations between dupilumab therapy, immune regulation, and a shift toward a more diverse skin-associated microbial community in rare genodermatoses such as NS.

This case report has several limitations inherent to its design as a single-case study. First, the findings are based on one patient and therefore cannot be generalized to the broader NS population. Second, the temporal association between dupilumab treatment, clinical improvement, and microbiome normalization does not establish a causal relationship. The observed microbial changes, including the predominance of *Pantoea*, may have been influenced by unmeasured environmental, hormonal, postpartum, or treatment-related factors. In addition, microbiome analysis was limited to serial observations without functional or mechanistic investigations, thereby preventing conclusions regarding the pathogenic role of specific microorganisms or their direct interaction with immune pathways. Finally, the absence of a control group and the limited longitudinal follow-up restrict the ability to determine the durability and reproducibility of the observed microbiome restoration over time.

## Patient perspective

After my first pregnancy, my skin became increasingly red and desquamated. I experienced constant itching, which had a big impact on my daily life and also made it difficult for me to sleep at night. These symptoms and the appearance of my skin impacted my confidence, and I would limit social interactions. Dupilumab completely changed my life; now, after 1 year of therapy, I have almost no itching; it no longer affects my sleep, and I can carry out all my daily activities.

## Data Availability

The datasets presented in this study can be found in online repositories. The names of the repository/repositories and accession number(s) can be found: https://www.ncbi.nlm.nih.gov/, PRJNA1378165.
